# Supplementary material for: Immunological history governs human stem cell memory CD4 heterogeneity via the Wnt signaling pathway
Source: Nat Commun. 2020 Feb 10;11:821. doi: 10.1038/s41467-020-14442-6 (PMC7010798; doi:10.1038/s41467-020-14442-6)
Supplement: Supplementary file 1 — Supplementary Information [file 41467_2020_14442_MOESM1_ESM.pdf]

Supplementary Information

Immunological History Governs Human Stem Cell Memory CD4 Heterogeneity Via The Wnt Signaling Pathway

Kared et al, Nature Communication, 2019

SUPPLEMENTARY FIGURE 1

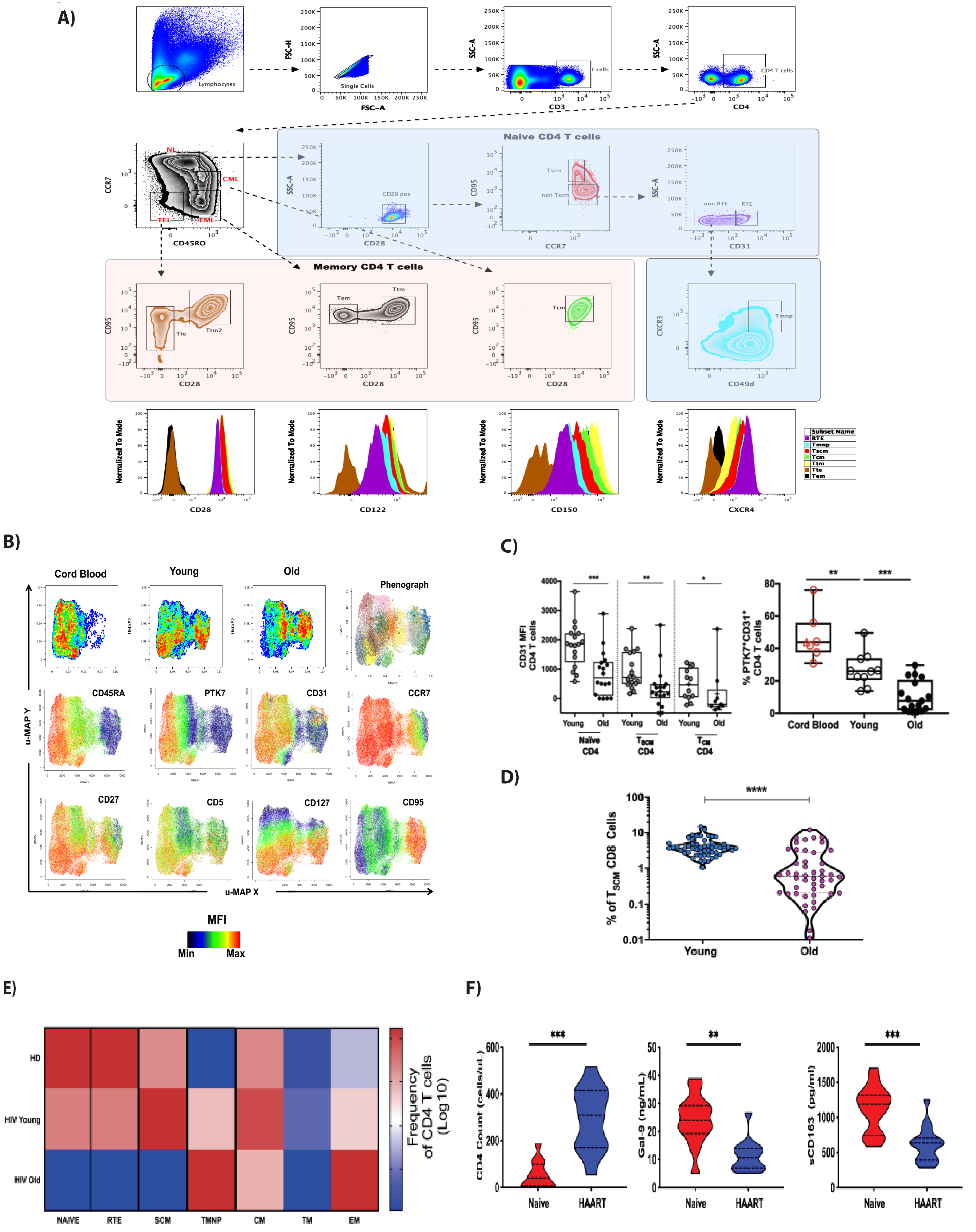

Supplementary Figures

Supplementary Figure 1 T_{SCM} CD4 cells heterogeneity

(A) Gating Strategy of T_{SCM} and CD4 T cell subsets from freshly isolated PBMCs. After the exclusion of doublets, lymphocytes were gated according to CD3 and CD4 T cells. CD45RO and CCR7 were used to define “naïve-like” (NL), “central memory-like”(CML), “effector memory-like (EML) and “terminal effector-like (TEL) subsets.. A bright expression of CD31 defined T_{RTE} in the non-T_{SCM} cells, while combined expression of CD49d and CXCR3 in non-RTE naïve CD4 T cells enabled the identification of T_{MNP}. Overlaid histograms of CD28, CD122, CD150 and CXCR4 summarized complementary markers useful to characterize the heterogeneity of CD4 T cell subsets.

(B) Representative clusters distribution of CD4 T cells during aging. Unbiased UMAP analysis was performed on total CD4 T cells from from cord blood, young or older donors. Clusters were automatically defined by phenograph. A four-color scale is used with black–blue indicating low expression values, green–yellow indicating intermediately expressed markers, and red representing highly expressed markers.

(C) Decreased of CD31 expression in CD4 T cells and of T_{RTE} frequencies in peripheral blood during aging. The Mean Fluorescence Intensity (MFI) of CD31 on naïve, T_{SCM} and T_{CM} CD4 cells was measured on frozen PBMCs. T_{RTE} ($CCR7^{+}CD45RO^{-}CD95^{-}CD27^{+}CD31^{+}PTK7^{+}$) were enumerated in frozen PBMCs. The MFI and T_{RTE} frequencies were compared during aging by a Mann-Whitney U test (* for $p<0.05$, ** for $p<0.01$ and *** for $p<0.001$). PBMCs from cord blood and T cells from foetal thymus (open triangle) were used as positive controls. Source data are provided as a Source Data file.

(D) Depletion of T_{SCM} CD8 cells during aging. Freshly isolated PBMCs were collected from Young (<35 years old, n=47) and Old participants (>65 years old, n=53) and stained for flow cytometry. Cell debris and dead cells were excluded from the analysis of gated lymphocytes based on scatter signals and live dead markers. Naïve cells were defined as $CCR7^{+}CD45RO^{-}CD27^{+}CD62L^{+}CD95^{-}$

and T_{SCM} as $CCR7^+CD45RO^-CD27^+CD62L^+CD95^+$ CD8 T cells. The frequency of T_{SCM} CD8 cells was measured on gated cell subsets and compared during aging by a Mann-Whitney U test (**** for p<0.0001).

(E) Distribution of naïve, T_{RTE}, T_{MNP}, T_{SCM}, T_{CM}, T_{TM} and T_{EM} CD4 cells during aging in HIV patients. A cold (blue) to hot (red) heat map represented the Log10-frequencies of CD4 T cells subsets in young and old aged HIV-infected patients. Source data are provided as a Source Data file.

(F) Inflammation and homeostasis of CD4 T cells during HIV therapy. CD4 T cells count was clinically measured. The concentrations of these molecules were measured before and 48 weeks after HAART-initiation.

SUPPLEMENTARY FIGURE 2

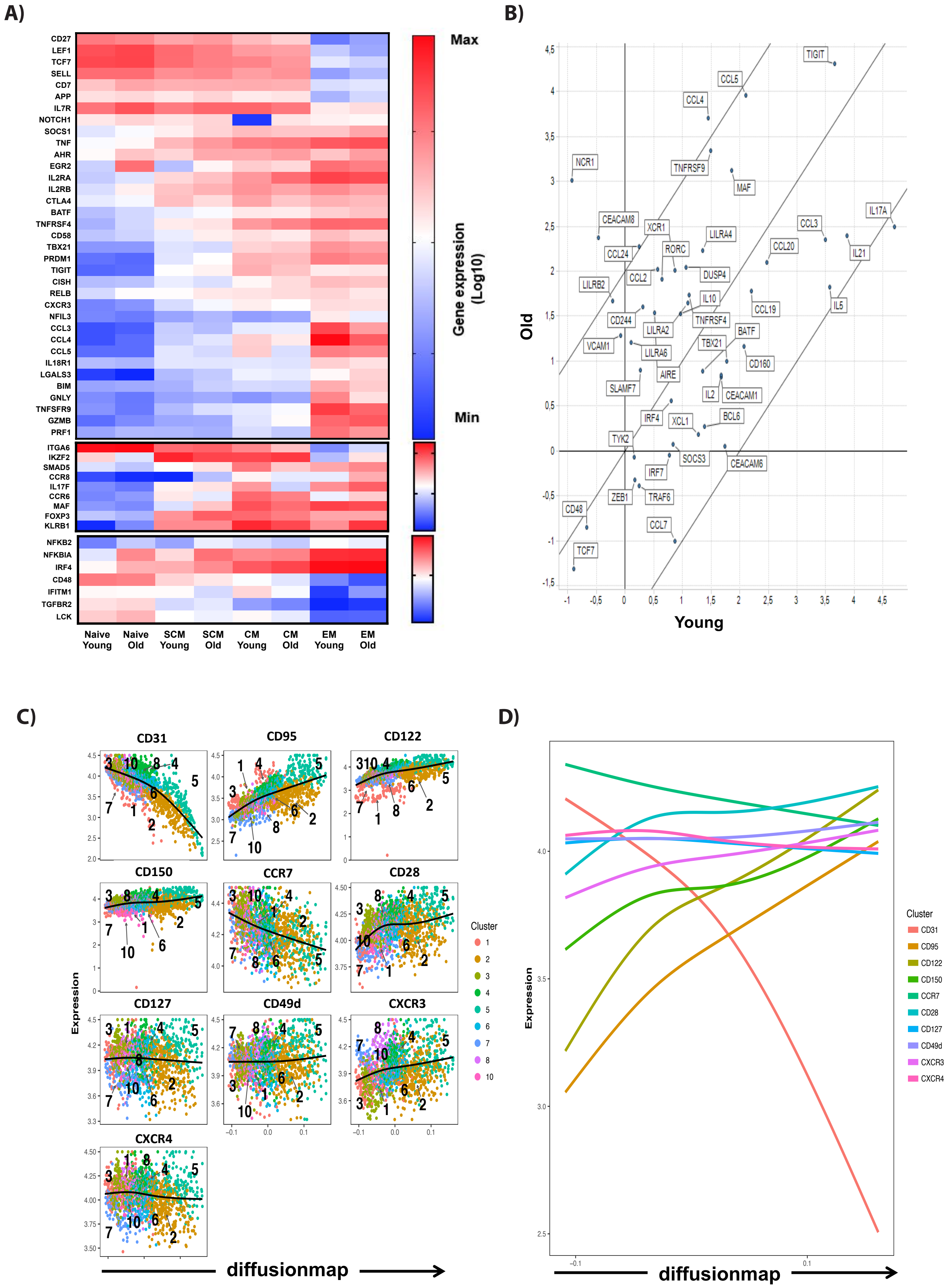

Supplementary Figure 2 Heterogeneity of T_{SCM} CD4 cells during aging

(A) Preservation of specific gene signature of CD4 T_{SCM} cells during aging. CD4 T cells (n=5 for all subsets by age except T_{EM}, n=3) were sorted and analyzed for their gene expression by Nanostring Source data are provided as a Source Data file.

(B) Differential transcriptional signature between naïve and T_{SCM} CD4 cells during aging. The preserved DEGs during aging (core signature of T_{SCM}) were located on the diagonal and the specific signature of T_{SCM} in young or older donors were along X- and Y-axis respectively.

(C) Progression of markers expression in naïve CD4 T cell populations. A diffusion map represented the progression of markers expression for each cluster of naïve CD4 T cells.

(D) Dynamic representation of naïve-associated markers. The differentiation of naïve CD4 T cells was constituted by the fine tune regulation of naïve- and memory-associated markers.

SUPPLEMENTARY FIGURE 3

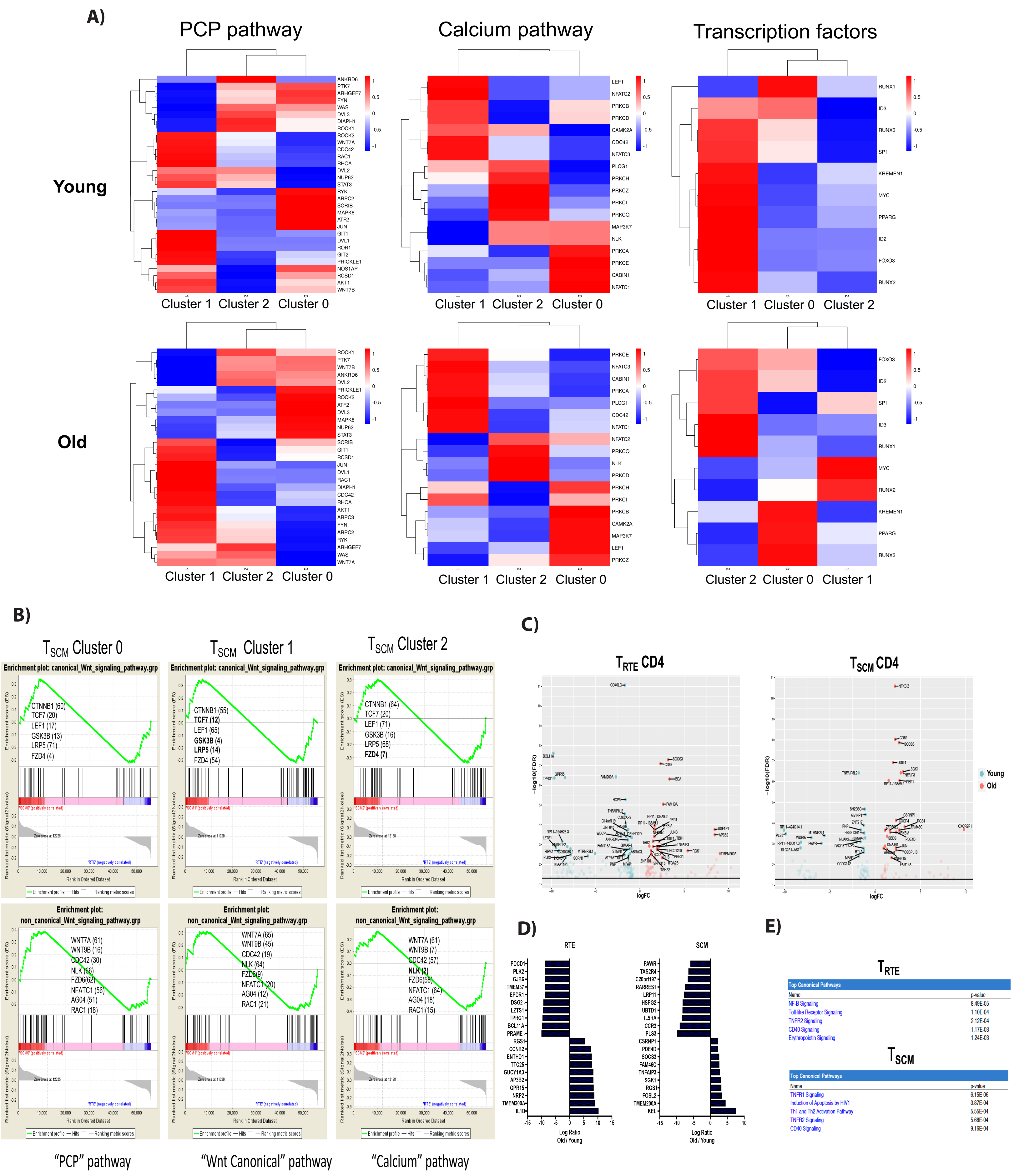

Supplementary Figure 3 Wnt pathway signature in T_{SCM} CD4 cells during aging

(A) Non-canonical Wnt pathway and transcription factors expression in the scRNAseq derived subsets of T_{SCM} CD4 cells during aging. The level of expression was normalized in order to compare the relative expression of each molecules in all clusters.

(B) Canonical and non-canonical Wnt signaling signature in CD4 T_{SCM} clusters from old donors. The enrichment of gene expression detected in CD4 T_{SCM} clusters for each pathway was calculated in comparison to T_{RTE} signature as detailed in figure 3E

(C) Specific age signature of CD4 T_{SCM} cells and T_{RTE}. Volcano plots represented the significant DEGs from RNAseq analysis in T_{RTE} and T_{SCM} detected during aging.

(D) Quantification of age signature in CD4 T_{RTE} and T_{SCM} cells from bulk RNAseq.

(E) Ingenuity Pathway analysis of RNAseq data of CD4 T_{SCM} and T_{RTE} cells from young and older donors.

SUPPLEMENTARY FIGURE 4

A)

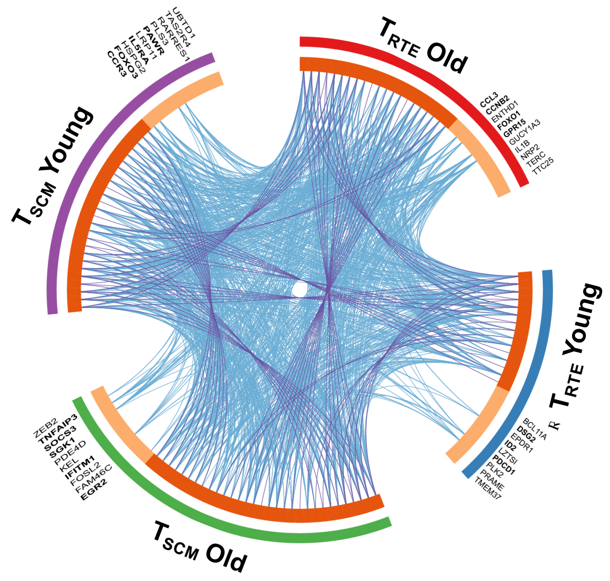

B)

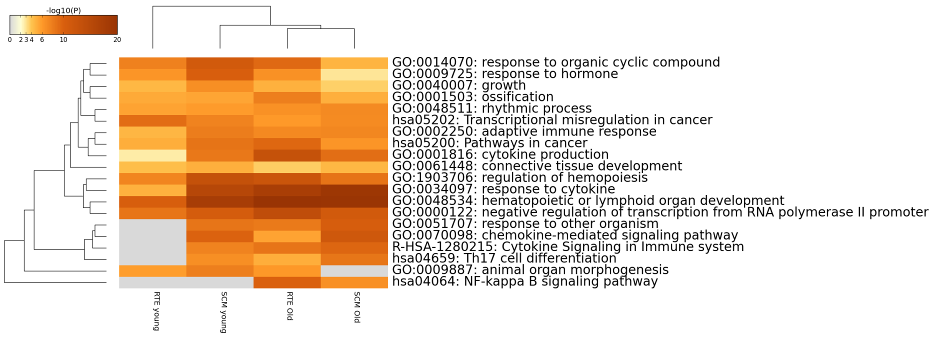

C)

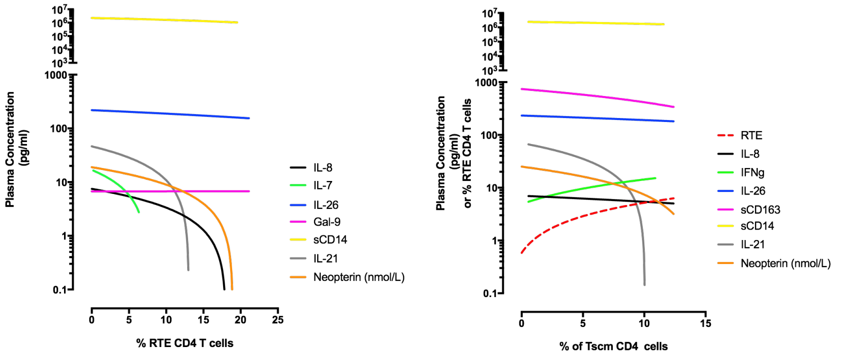

D)

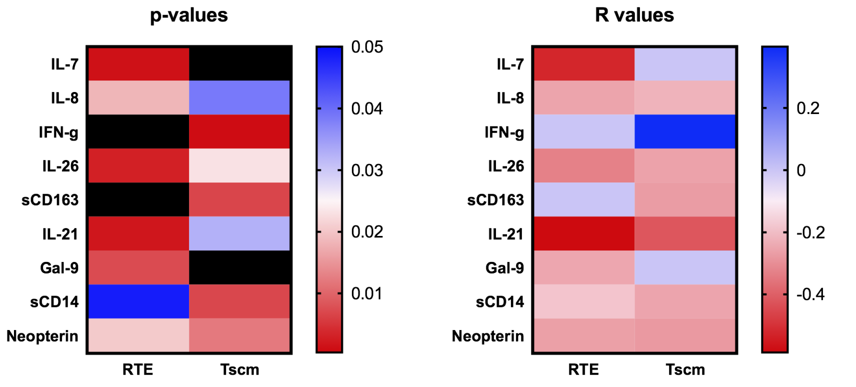

E)

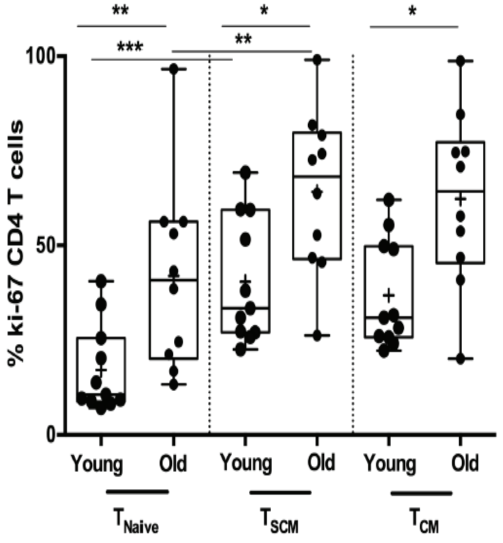

Supplementary Figure 4 Inflammation and homeostasis of T_{SCM} CD4 cells

(A) Redundancy of age signature between CD4 T_{RTE} and T_{SCM} cells. The specific signatures of T_{RTE} and T_{SCM} CD4 cells were overlaid with Metascape. The specific genes of each subset during aging were labeled in bold.

(B) Canonical pathway analysis of Age signature in CD4 T_{RTE} and T_{SCM} cells. The DEGs between T_{RTE} and T_{SCM} CD4 cells during aging were analyzed by Ingenuity pathway.

(C) Inflammation and homeostasis of T_{SCM} CD4 cells. Inflammatory and homeostatic cytokines were measured in the plasma of the Asian HIV cohort by Luminex or Elisa. The concentrations of inflammatory molecules were correlated with the frequencies of T_{RTE} and T_{SCM} CD4 T cells (Spearman's rank order test).

(D) Two individual heat maps represented the significance and correlation's coefficient between the amount of inflammatory markers and the frequency of CD4 T_{SCM} cells. The cold (blue) to hot (red) p-values represent respectively the weakest to the strongest correlation. The black box indicated the absence of association between inflammatory molecules and CD4 T cell subsets. Source data are provided as a Source Data file.

(E) Increased cycling activity in naïve and T_{SCM} CD4 cells during aging. The proliferation marker, Ki-67 was intracellular measured in gated naïve, T_{CM} and T_{SCM} CD4 cells. The statistical analysis was performed on paired samples to compare T cell subsets (Wilcoxon signed-rank test) or unpaired samples (Mann-Whitney U test; n=11 and =10 for young and older donors respectively) to evaluate the influence of aging on Ki-67 acquisition (* for $p<0.05$, ** for $p<0.01$ and *** for $p<0.001$).

SUPPLEMENTARY FIGURE 5

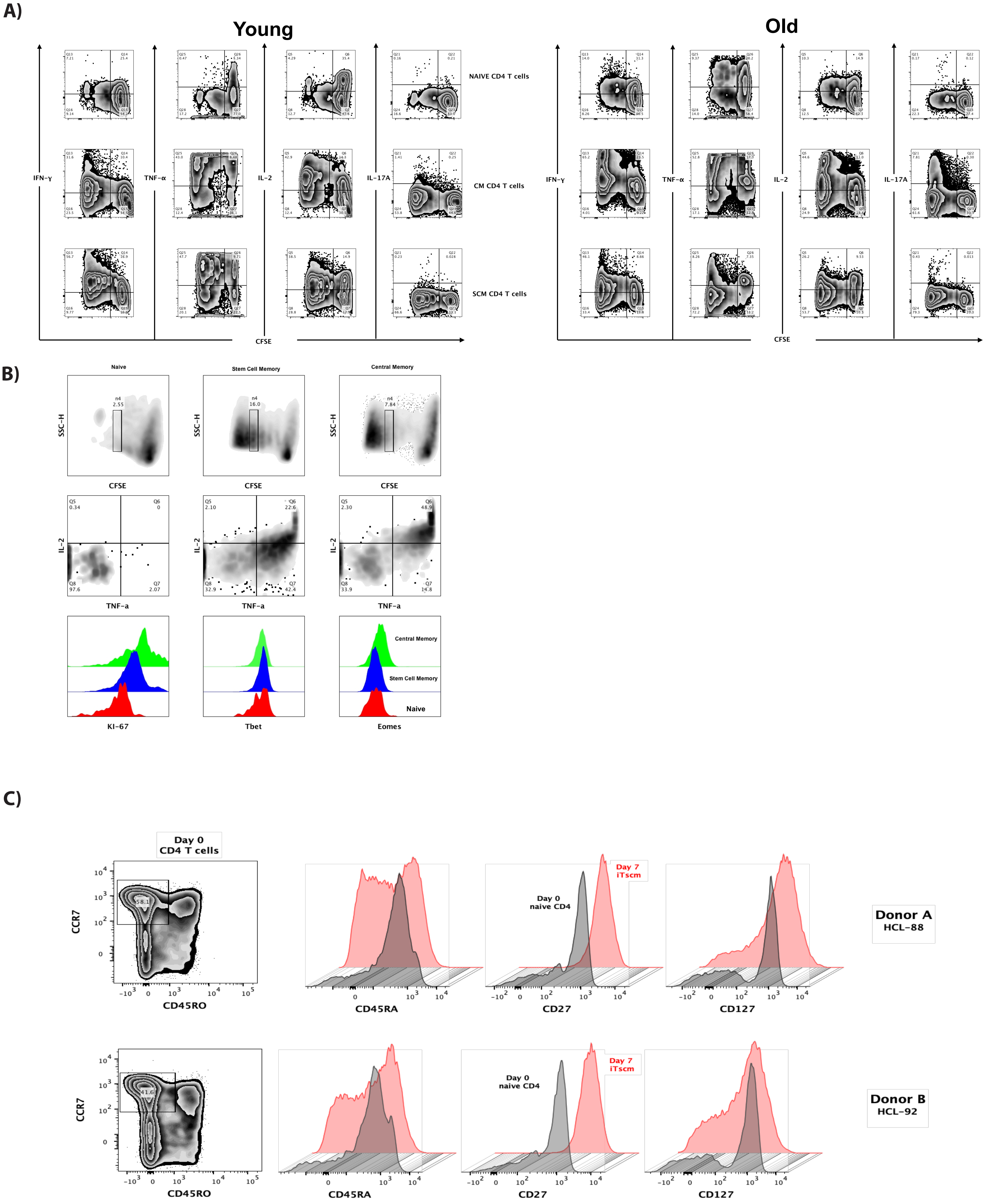

Supplementary Figure 5 Functions of T_{SCM} CD4 cells during aging

(A) Preserved characteristics of proliferating T_{SCM} CD4 cells. Sorted naïve, T_{SCM} and T_{CM} were CFSE-stained and stimulated through their TCR during 5 days. Cells with similar proliferating history (4 divisions) were compared for their secretion of cytokines in response to PMA/ Ionomycin re-stimulation and their expression of Ki-67 and transcription factors (T-bet/ Eomes).

(B) Decrease of IL-2 and TNF secretion by proliferating T_{SCM} during aging. Similarly to (A), cells subsets from young and old donors proliferated in response to anti-CD3/CD28 micro beads and were polyclonally re stimulated at day 5 to assess cytokines secretion.

(C) Phenotype of transferred iT_{SCM} CD4 cells. Purified naïve CD4 T cells were stimulated in vitro during 7 days in presence of IL-7 and IL-15. The phenotype was performed on gated naïve CD4 T cells from total PBMCs at baseline and on induced T_{SCM} CD4 cells at day 7. The overlaid expression of CD45RA, CD27 and CD127 was represented for two independent donors.

SUPPLEMENTARY FIGURE 6

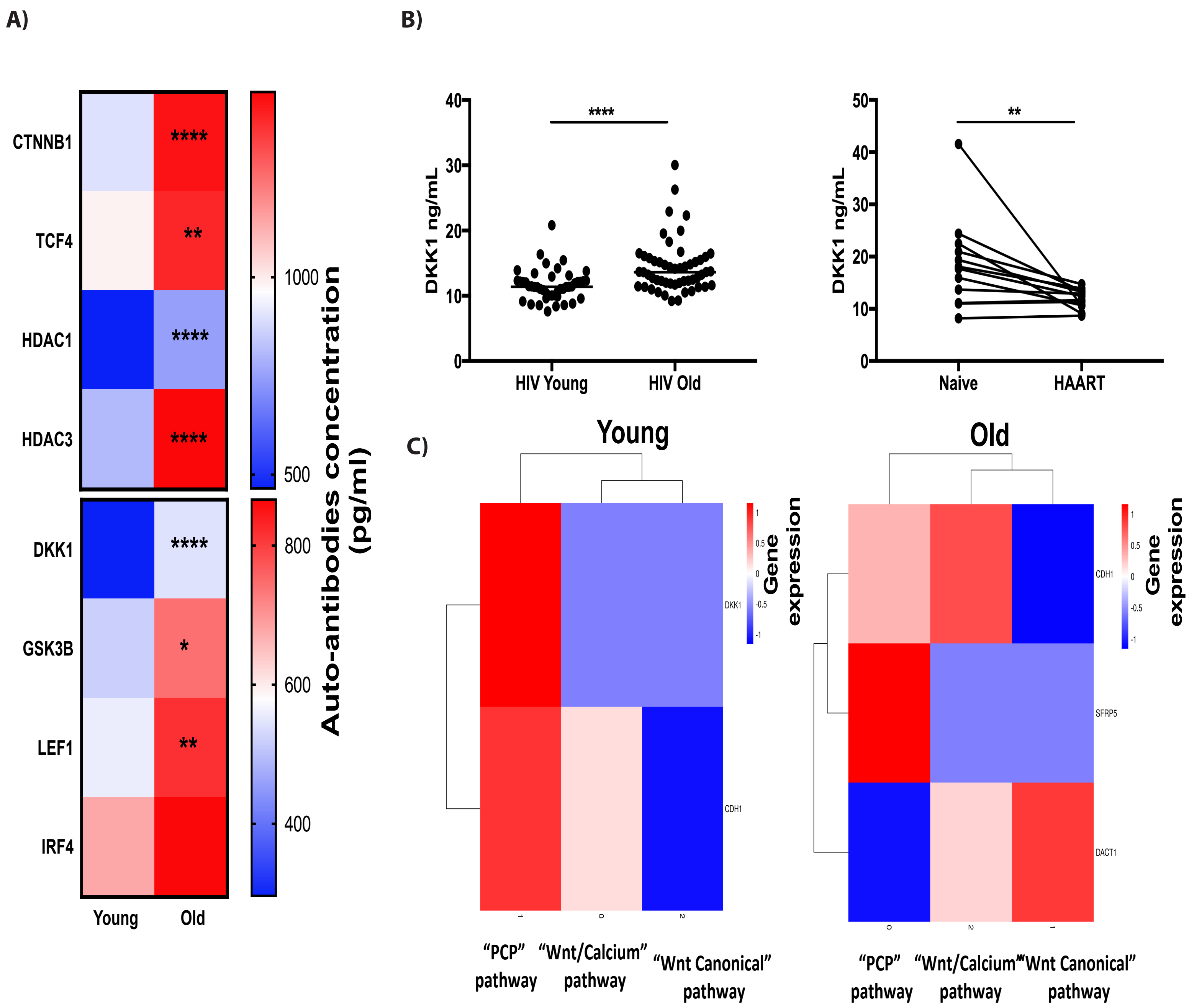

Supplementary Figure 6 Activation of Wnt/β-Catenin pathway during aging

(A) Increased concentration of autoantibodies against constitutive molecules of Wnt/β-Catenin signaling pathway during aging. The plasmatic levels of analytes in young and older donors were measured by Elisa (n= 93 and n= 60 respectively). The statistical analysis was performed on unpaired samples (Mann-Whitney U test) (* for $p<0.05$, ** for $p<0.01$ and **** for $p<0.0001$). Source data are provided as a Source Data file.

(B) Systemic DKK1 increased with age during HIV infection and is modulated by HIV therapy. The concentration of the Wnt/β-Catenin inhibitor was measured in the plasma of young and old HIV-infected patients or before and 48 weeks after the initiation of the anti-retroviral therapy. The statistical analysis was performed on paired samples to compare DKK1 concentrations (Wilcoxon signed-rank test for the longitudinal follow-up of non-treated versus ART patients) or unpaired samples (Mann-Whitney U test for HIV young versus HIV old) (** for $p<0.01$ and **** for $p<0.0001$). Source data are provided as a Source Data file.

(C) Inhibitors of Wnt Signaling pathway in T_{SCM} CD4 cells. The relative gene expression of Wnt signaling's natural inhibitors was quantified from scRNAseq data. The results were visualized by a cold to hot heatmap.

SUPPLEMENTARY FIGURE 7

A)

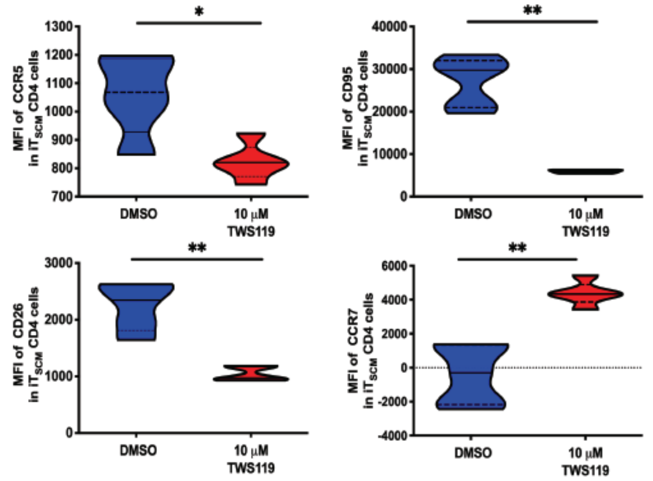

B)

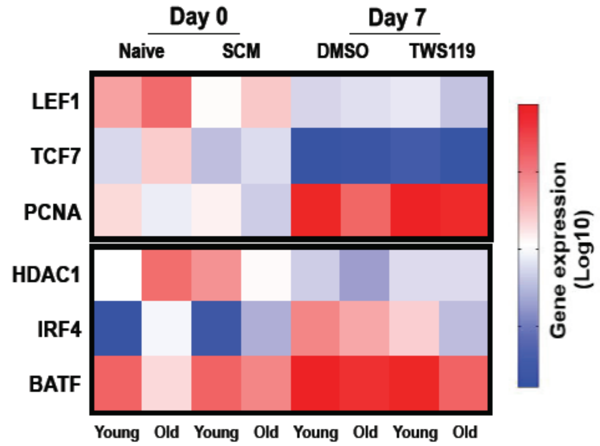

C)

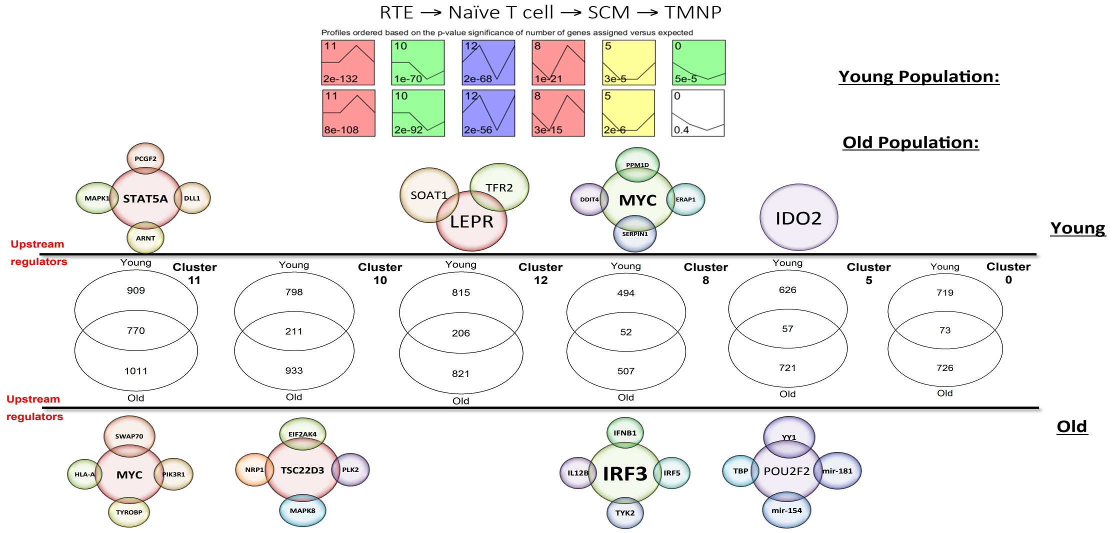

D)

Top Canonical Pathways

Name Young Donors	p-value	Cluster 11	Name Old Donors	p-value
Apoptosis Signaling	3.62E-05		Sirtuin Signaling Pathway	9.22E-05
Death Receptor Signaling	1.32E-04		Oxidative Phosphorylation	2.65E-04
CDP-diacylglycerol Biosynthesis I	3.51E-04		Remodeling of Epithelial Adherens Junctions	5.80E-04
Phosphatidylglycerol Biosynthesis II (Non-plastidic)	5.85E-04		RhoA Signaling	9.69E-04
Triacylglycerol Biosynthesis	7.28E-04		Unfolded protein response	1.23E-03

Name Young Donors	p-value	Cluster 10	Name Old Donors	p-value
Antiproliferative Role of TOB in T Cell Signaling	4.08E-02		EIF2 Signaling	3.37E-03
Circadian Rhythm Signaling	5.85E-02		Regulation of eIF4 and p70S6K Signaling	2.03E-02
L-carnitine Biosynthesis	5.85E-02		Systemic Lupus Erythematosus Signaling	2.30E-02
Taurine Biosynthesis	5.85E-02		Hereditary Breast Cancer Signaling	3.10E-02
L-glutamine Biosynthesis II (tRNA-dependent)	5.85E-02			

Name Young Donors	p-value	Cluster 12	Name Old Donors	p-value
EIF2 Signaling 8.9 % 17/191	1.26E-04		mTOR Signaling	3.18E-03
Regulation of eIF4 and p70S6K Signaling	4.80E-04		Mechanisms of Viral Exit from Host Cells	4.55E-03
tRNA Charging	9.97E-04		EIF2 Signaling	6.04E-03
UDP-N-acetyl-D-glucosamine Biosynthesis II	1.39E-02		Regulation of eIF4 and p70S6K Signaling	1.05E-02
Valine Degradation I	1.82E-02		Macropinocytosis Signaling	2.29E-02

Name Young Donors	p-value	Cluster 8	Name Old Donors	p-value
Regulation of Actin-based Motility by Rho	1.30E-05		Actin Cytoskeleton Signaling	1.65E-03
TCA Cycle II (Eukaryotic)	1.31E-05		TNFR1 Signaling	1.71E-03
Aspartate Degradation II	1.79E-04		Sumoylation Pathway 9.2 % 8/87	3.05E-03
Protein Ubiquitination Pathway	2.03E-04		Regulation of Actin-based Motility by Rho	3.16E-03
RhoA Signaling	2.50E-04		Glutathione Redox Reactions II	4.55E-03

Name Young Donors	p-value	Cluster 5	Name Old Donors	p-value
Systemic Lupus Erythematosus Signaling	5.05E-04		Tetrapyrrole Biosynthesis II	3.77E-03
EIF2 Signaling	8.72E-04		Glycogen Biosynthesis II (from UDP-D-Glucose)	6.18E-03
Serine Biosynthesis	4.41E-03		DNA damage-induced 14-3-3 Signaling	1.01E-02
Regulation of eIF4 and p70S6K Signaling	5.26E-03		Estrogen Receptor Signaling	1.13E-02
Superpathway of Serine and Glycine Biosynthesis I	1.06E-02		Heme Biosynthesis II	1.65E-02

E)

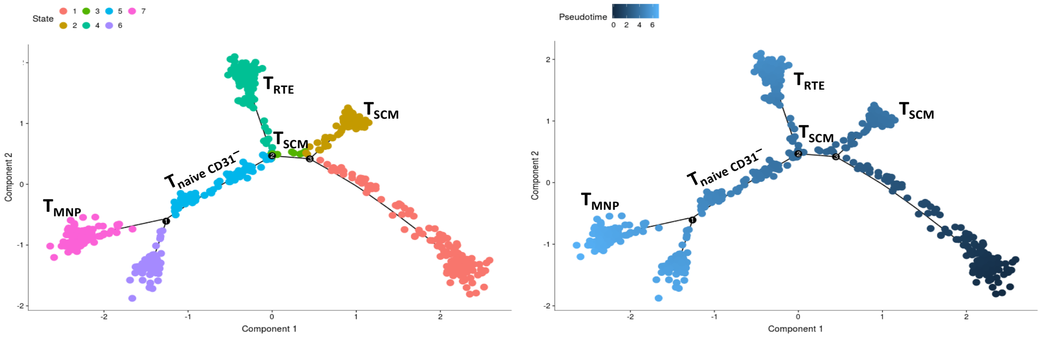

Supplementary Figure 7 Characteristics of iT$_{SCM}$ CD4 cells during aging

(A) Phenotype of iT$_{SCM}$ CD4 cells in aged donors in response to high concentration of TWS119. CD95, CCR5, CD26 and CCR7 expression was quantified by flow cytometry at day 8 in DMSO- or TWS119-stimulated naïve ($CCR7^{+}CD45RO^{-}CD27^{+}CD95^{-}$) CD4 T cells. The statistical analysis was performed on paired samples (n=5) to compare MFI of surface markers between DMSO- and TWS119-stimulated naïve CD4 T cells (paired sample T-Test)(* for $p<0.05$ and ** for $p<0.01$). Source data are provided as a Source Data file.

(B) Transcriptional profile of iT$_{SCM}$ CD4 cells during aging. The mRNAs of sorted subsets were extracted directly ex-vivo or after 8 days of stimulation with DMSO or TWS119 for naïve CD4 T cells. The transcripts of genes coding for constitutive molecules of Wnt/β-Catenin signaling were quantified by quantitative PCR. The Log10 values of gene expression were visualized by a cold to hot heatmap.

(C) Modelisation of naïve CD4 T cells differentiation and regulation during aging. STEM analysis of bulk naïve subsets CD4 T cells RNAseq data. This hypothetical trajectory of differentiation was associated with several patterns of gene dynamic (classified as Clusters 11, 10, 12, 8, 5 and 0). They were hierarchically organized according to their significance. The genes of these clusters were either preserved during aging either specific to Young or Old donors. IPA revealed that the age-specific genes in each cluster were differentially regulated.

(D) Canonical pathway analysis of cluster signature in naïve CD4 T cell subsets during aging. The analysis of each cluster was performed by Ingenuity pathway in each group of age and indicated a “metabolic” signature associated with aging.

(E) Modelisation of naïve CD4 T cells differentiation and regulation during aging. Monocle analysis of single-cell RNA-seq data from naïve CD4 T cell subset. Protein (CD28, CD31,

CCR7, CD95, CD122, and CD127) and mRNA (PTK7, CD31) expression were overlaid on different branches to determine T cell subsets. This hypothetical trajectory of differentiation was associated with pseudotime and represented the kinetic of single-cell gene expression.

SUPPLEMENTARY FIGURE 8

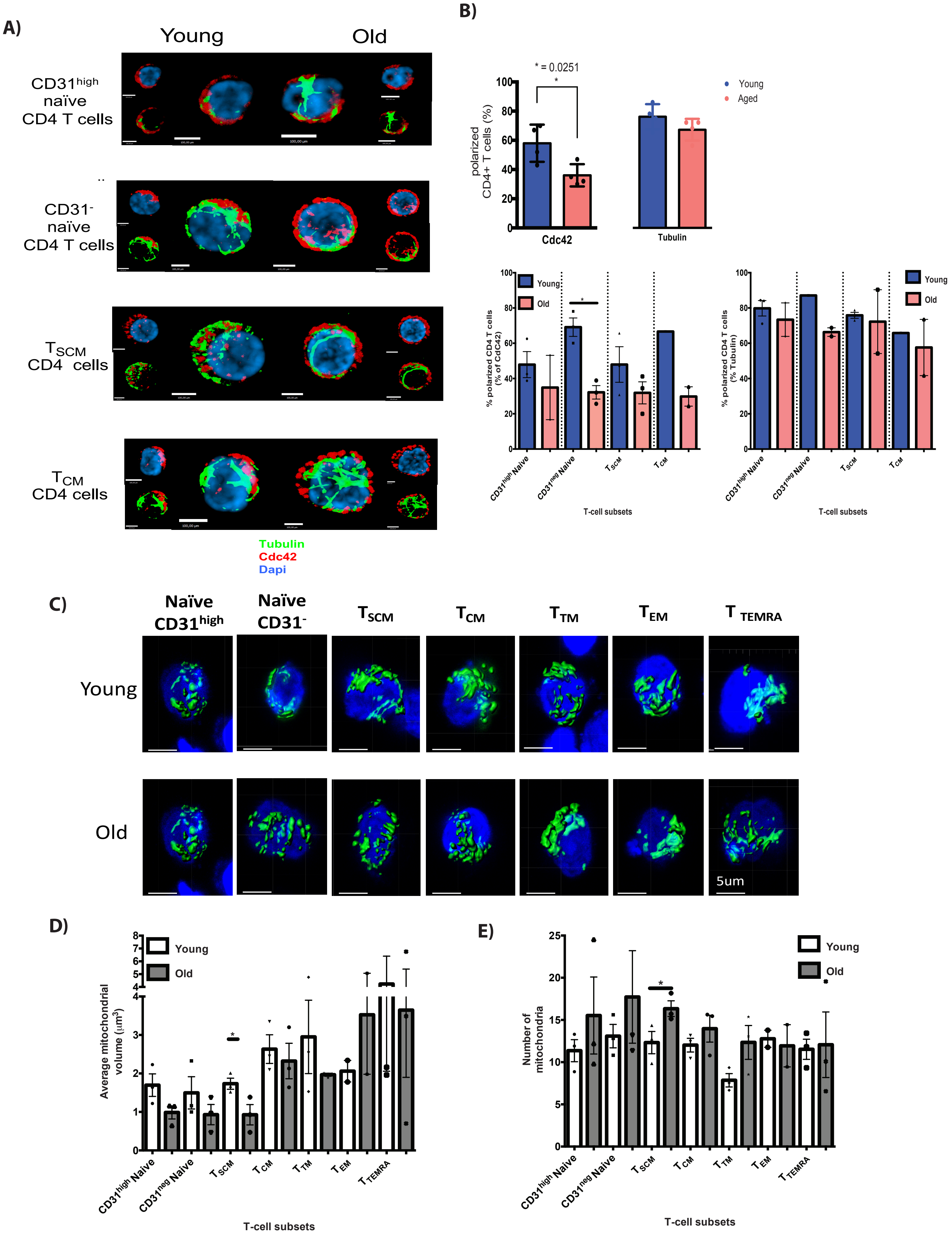

Supplementary Figure 8 Polarization and metabolism of T_{SCM} CD4 cells

(A) Polarization of CD4 T cells during aging. The architecture of immune T cells during aging was analysed in sorted CD4 T cells by confocal microscopy experiments. T cell subsets were sorted as naïve ($CCR7^+CD45RO^-CD27^+CD31^-CD95^-$), T_{RTE} ($CCR7^+CD45RO^-CD27^+CD31^{high}CD95^-$), T_{SCM} ($CCR7^+CD45RO^-CD27^+CD95^+$) and T_{CM} ($CCR7^+CD45RO^+CD27^+CD95^+$) CD4 T cells from young or old individuals (n=5 per group). The lymphocytes were TCR-stimulated with a-CD3/CD28 microbeads during 16 hours. The T cells were fixed and permeabilized, before intra-cellular staining for DAPI (nucleus), Tubulin and Cdc42.

(B) Decreased polarization of naïve CD4 T cells during aging. The reorganization of cells architecture induced by TCR activation was measured by confocal microscopy. Quantification of T cell polarization in total and CD4 T cell subsets depended on donor 's age. Results are presented as mean±S.D.

(C) Metabolism and Mitochondrial contents in CD4 T cells during aging. Representative clichés of sorted CD4 T cell subsets isolated in young and old donors. Following the flow cytometer sorting, T cells rested overnight at 37C. CD4 subsets were intra-cellular stained to evaluate the number and volume of mitochondria by microscopy.

(D) Decreased of mitochondrial volume during aging. The volume of mitochondria in 15-20 individual cells was automatically measured by the imaging software (Imaris). Three donors in each age group were analyzed in 3 independent experiments. Results are presented as mean±S.D. Source data are provided as a Source Data file.

(E) Increased of mitochondrial number during aging in CD4 T_{SCM} cells.

The number of mitochondria in 15-20 individual cells was automatically measured by the imaging software (Imaris). Three donors in each age group were analyzed in 3 independent experiments. Results are presented as mean±S.D.

Supplementary Tables

Sup Table 1: Phenotype of CD4 T cells subsets

Name	Abbreviation	Phenotype	notes
Recent Thymic Emigrants	T_{RTE}	$CCR7^{+}CD45RO^{-}CD27^{+}CD28^{+}CD62L^{+}CD122^{-}CD95^{-}CD31^{high}PTK7^{+}$	CD62L only used in freshly isolated cells
Naive	$T_{NAÏVE}$	$CCR7^{+}CD45RO^{-}CD27^{+}CD28^{+}CD62L^{+}CD122^{-}CD95^{-}CD31^{dim}$	CD62L only used in freshly isolated cells
Memory T cells with a Naïve Phenotype	T_{MNP}	$CCR7^{+}CD45RO^{-}CD27^{+}CD28^{+}CD122^{-}CD95^{-}CD31^{-}CD49d^{high}CXCR3^{+}$	
Stem Cell Memory	T_{SCM}	$CCR7^{+}CD45RO^{-}CD27^{+}CD62L^{+}CD122^{+}CD95^{+}$	CD62L only used in freshly isolated cells
Virtual Memory	T_{VM}	$CCR7^{+}CD45RO^{-}CD27^{-}CD5^{-}CD95^{-}$	Better identification with $CD122^{+}CD127^{+}$<br><br>$PanKIR^{+}NKG2A^{+}Eomes^{+}$
Central Memory	T_{CM}	$CCR7^{+}CD45RO^{+}CD27^{+}CD28^{+}CD95^{+}$	
Transitional Memory	T_{TM}	$CCR7^{-}CD45RO^{-/+}CD27^{-/+}CD28^{+}CD95^{+}$	TM1/2 could be distinguish based on CD45RO and CD27 expression
Effector Memory	T_{EM}	$CCR7^{-}CD45RO^{+}CD27^{-}CD28^{-}CD95^{+}$	
Terminal Effector Memory RA	T_{EMRA}	$CCR7^{-}CD45RO^{-}CD45RA^{+}CD27^{-}CD28^{-}CD95^{+}$	

Sup Table 2: Clinical characteristics of aging cohort

	Age (Median ± SD)	Gender (n Male/ Total)	**Serology: % positive donors (Median IgG value, U/mL)** H Pylori IgG	EBV EBNA1 IgG	VZV IgG	HSV1 IgG	HSV2 IgG	CHKN IgG	Dengue IgG	CMV IgG
**Young**	22 ± 5	31/62	5 % (4)	80 % (54)	87 % (886)	35% (21)	0% (2.9)	9% (5)	18% (1)	38% (8)
**Old**	68 ± 9	79/219	30 % (12)	98 % (34)	96% (761)	86% (78)	19% (3.3)	1% (2)	87% (32)	97% (781)
		**IgG concentration** Mann-Whitney U test	****	****	**N.S.**	****	***	****	****	****
		Negative	<35	<2.5	<50	<20	<20	<9	<9	<25
		Borderline range	35-50	2.5-3	50-100	20-30	20-30	9-11	9-11	25-40
		Positive	>50	>3	>100	>30	>30	>11	>11	>40
		Units	U/ml	U/ml	mIU/ml	U/ml	U/ml	Stand. Units	Panbio Units	PEI-U/mL
		Brand	Virion\serion	Virion \serion	Virion \serion	Virion\serion	Virion\serion	Abcam	Panbio	Virion\serion
		Detection range	5-500	1-200	15-2000	10-1000	10-1000	N.A.	N.A.	10-2000

S.D.: Standard Deviation

N.A.: Not Available

Sup Table 3: List of flow cytometry antibodies

	Name	Clone	Company	Dilution
	CD3	UCHT1	BD Biosciences	1/50
	CD4	OKT4	BD Biosciences	1/50
	CD5	UCHT2	BD Biosciences	1/50
	CD8	OKT8	BD Biosciences	1/50
	CD25	MA251	Biolegend	1/25
	CD27	L128	BD Biosciences	1/50
	CD28	CD28.2	BD Biosciences	1/100
	CD31	WM-59	BD Biosciences	1/25
	CD38	HIT2	BD Biosciences	1/50
	CD45RA	HI100	BD Biosciences	1/100
	CD45RO	UCHL1	Biolegend	1/50
	CD49d	9F10	BD Biosciences	1/25
	CD56	HCD56	BD Biosciences	1/50
	CD57	HNK-1	Biolegend	1/100
	CD62L	DREG-56	Biolegend	1/50
**Surface**	CD95	DX2	BD Biosciences	1/50
**Staining**	CD122	Mik-β2	BD Biosciences	1/25
	CD127	HIL-7R-M21	BD Biosciences	1/50
	CD150	A12(7D4)	Biolegend	1/50
	CD161	HP-3G10	Biolegend	1/50
	CXCR3	G025H7	Biolegend	1/25
	CXCR4	12G5	Biolegend	1/50
	CXCR5	J252D4	Biolegend	1/50
	CCR5	J418F1	Biolegend	1/25
	CCR6	11A9	BD Biosciences	1/50
	CCR7	G043H7	Biolegend	1/25
	PTK7	CCK4	Miltenyi	1/25
	Vα7.2	3C10	BD Biosciences	1/50
	PAN GD	11F2	BD Biosciences	1/50
	KLRG1	2F12/KLRG1	eBioscience	1/25
	CD352	REA 339	Miltenyi	1/25
	CRTH2	BM16	Biolegend	1/50
	HLA-DR	L243	BD Biosciences	1/100
	IFN-g	4S.B3	Biolegend	1/50
	TNF	MAb11	BD Biosciences	1/50
	IL-2	MQ1-17H12	Biolegend	1/50
**Intra-cellular**	IL-17A	64DEC17	eBioscience	1/25
**Intra-nuclear**	TCF-1	7F11A10	Biolegend	1/25
**Staining**	T-bet	4B10	eBioscience	1/50
	CTLA-4	14D3	BD Biosciences	1/25
	FOXP3	PCH101	eBioscience	1/25
	KI-67	Ki-67	BD Biosciences	1/25
	Eomes	WD1928	eBioscience	1/50
