# Supplementary material for: Immunological history governs human stem cell memory CD4 heterogeneity via the Wnt signaling pathway
Source: Nat Commun. 2020 Feb 10;11:821. doi: 10.1038/s41467-020-14442-6 (PMC7010798; doi:10.1038/s41467-020-14442-6)
Supplement: Supplementary file 3 — Reporting Summary [file 41467_2020_14442_MOESM3_ESM.pdf]

nature research

Corresponding author(s): KARED HASSEN

Last updated by author(s): Dec 17, 2019

# Reporting Summary

Nature Research wishes to improve the reproducibility of the work that we publish. This form provides structure for consistency and transparency in reporting. For further information on Nature Research policies, see Authors & Referees and the Editorial Policy Checklist .

## Statistics

For all statistical analyses, confirm that the following items are present in the figure legend, table legend, main text, or Methods section.

| n/a | Confirmed | |
|---|---|---|
| ☐ | ☒ | The exact sample size (*n*) for each experimental group/condition, given as a discrete number and unit of measurement |
| ☐ | ☒ | A statement on whether measurements were taken from distinct samples or whether the same sample was measured repeatedly |
| ☐ | ☒ | The statistical test(s) used AND whether they are one- or two-sided<br>*Only common tests should be described solely by name; describe more complex techniques in the Methods section.* |
| ☐ | ☒ | A description of all covariates tested |
| ☐ | ☒ | A description of any assumptions or corrections, such as tests of normality and adjustment for multiple comparisons |
| ☐ | ☒ | A full description of the statistical parameters including central tendency (e.g. means) or other basic estimates (e.g. regression coefficient) AND variation (e.g. standard deviation) or associated estimates of uncertainty (e.g. confidence intervals) |
| ☐ | ☒ | For null hypothesis testing, the test statistic (e.g. *F*, *t*, *r*) with confidence intervals, effect sizes, degrees of freedom and *P* value noted<br>*Give P values as exact values whenever suitable.* |
| ☒ | ☐ | For Bayesian analysis, information on the choice of priors and Markov chain Monte Carlo settings |
| ☐ | ☒ | For hierarchical and complex designs, identification of the appropriate level for tests and full reporting of outcomes |
| ☐ | ☒ | Estimates of effect sizes (e.g. Cohen's *d*, Pearson's *r*), indicating how they were calculated |

*Our web collection on statistics for biologists contains articles on many of the points above.*

## Software and code

Policy information about availability of computer code

| | |
|---|---|
| Data collection | Microsoft Excel, GraphPad Prism |
| Data analysis | Gene expression: Metascape, MiXCR, Monocle, Seurat R package (https://github.com/satijalab/seurat).<br>Gene Set Enrichment Analysis (GSEA v3.0). The pathways were retrieved from AMIGO2 website |

For manuscripts utilizing custom algorithms or software that are central to the research but not yet described in published literature, software must be made available to editors/reviewers. We strongly encourage code deposition in a community repository (e.g. GitHub). See the Nature Research guidelines for submitting code & software for further information.

## Data

Policy information about availability of data

All manuscripts must include a data availability statement. This statement should provide the following information, where applicable:

- Accession codes, unique identifiers, or web links for publicly available datasets
- A list of figures that have associated raw data
- A description of any restrictions on data availability

The data that support the findings of this study are available from the corresponding author upon reasonable request

# Field-specific reporting

Please select the one below that is the best fit for your research. If you are not sure, read the appropriate sections before making your selection.

# Life sciences study design

All studies must disclose on these points even when the disclosure is negative.

| | |
|---|---|
| Sample size | Sample size were chosen according to sample availability. PBMCs from young (NUS cohort), older donors (SLAS cohort) and HIV-infected patients (University of Malaya) were collected over the last five years. Fresh blood from young, older and HAART treated HIV-infected patients were also included in this study. Plasma were collected over the last five years from the same cohorts and cryo-preserved. Among all samples analysed, we have included data from 99 young and 913 older donors for the plasma and 62 or 219 samples for fresh blood of young and older donors respectively. |
| Data exclusions | Poor quality samples was defined by a low cell number (bad recovery) and/or high cell dead number, and were not included in the study. |
| Replication | We reproduced our analysis using freshly isolated PBMC from 23 young and 78 older donors. Our HIV data were reproduced in another HIV cohort (Sevilla, Dr. Ezequiel Ruiz-Mateos) but were not included in this manuscript. |
| Randomization | The samples were randomly organized for the Elisa and Luminex experiments. Only longitudinal samples were run on the same plate. |
| Blinding | The investigators were blinded to group allocation during data collection and/or analysis. |

# Reporting for specific materials, systems and methods

We require information from authors about some types of materials, experimental systems and methods used in many studies. Here, indicate whether each material, system or method listed is relevant to your study. If you are not sure if a list item applies to your research, read the appropriate section before selecting a response.

## Materials & experimental systems

| n/a | Involved in the study | |
|---|---|---|
| ☐ | ☒ | Antibodies |
| ☒ | ☐ | Eukaryotic cell lines |
| ☒ | ☐ | Palaeontology |
| ☒ | ☐ | Animals and other organisms |
| ☐ | ☒ | Human research participants |
| ☒ | ☐ | Clinical data |

## Methods

| n/a | Involved in the study | |
|---|---|---|
| ☒ | ☐ | ChIP-seq |
| ☐ | ☒ | Flow cytometry |
| ☒ | ☐ | MRI-based neuroimaging |

# Antibodies

| | |
|---|---|
| Antibodies used | See extended Table2 for details. Antibody name (Clone, manufacturer)<br>CD3(UCHT1, BD Biosciences), CD4 (OKT4, BD Biosciences), CD5 (UCHT2, BD Biosciences), CD8 (OKT8, BD BiosciencesCD25MA251BiolegendCD27L128BD BiosciencesCD28CD28.2BD BiosciencesCD31WM-59BD BiosciencesCD38HIT2BD Biosciences), CD45RA (HI100, BD Biosciences), CD45RO (UCHL1, Biolegend), CD49d (9F10, BD Biosciences), CD56 (HCD56, BD Biosciences), CD57(HNK-1, Biolegend), CD62L (DREG-56, Biolegend), CD95(DX2, BD Biosciences), CD122(Mik-β2, BD Biosciences), CD127(HIL-7R-M21, BD Biosciences), CD150 (A12(7D4)), Biolegend), CD161(HP-3G10, Biolegend), CXCR3(G025H7, Biolegend), CXCR4 (12G5, Biolegend), CXCR5(J252D4, Biolegend), CCR5(J418F1, Biolegend), CCR6(11A9, BD Biosciences), CCR7 (G043H7, Biolegend), PTK7(CCK4, Miltenyi), Va7.2 (3C10, BD Biosciences), PAN GD(11F2, BD Biosciences), KLRG1(2F1/KLRG1, eBioscience), CRTH2 (BM16, Biolegend), HLA-DR(L243, BD Biosciences), IFN-g (4S.B3, Biolegend), TNF-a (MAb11, BD Biosciences), IL-2 (MQ1-17H12, Biolegend), IL-17A(64DEC17, eBioscience), T-bet (4B10, eBioscience), CTLA-4 (14D3, BD Biosciences), FOXP3 (PCH101, eBioscience), KI-67(Ki-67, BD Biosciences), Eomes (WD1928, eBioscience). |
| Validation | All antibodies have been validated by the manufacturer. |

## Human research participants

Policy information about studies involving human research participants

| | |
|---|---|
| Population characteristics | Population characteristics have been recently published (Kared et al, 2018, Frontiers Immunology; and Lu et al, 2016, Oncotarget).<br>Additional information can be provided if needed |
| Recruitment | All participants gave informed and written consent.<br>Data on HIV-specific characteristics including HIV RNA, CD4 T cell counts, anti-retroviral drug history and history of co-infections were obtained from patient medical records. |
| Ethics oversight | The study has been approved by the National University of Singapore-Institutional Review Board 04–140.<br>The study has been approved by the Ethics Committee of the NUS-IRB 09-256.<br>The study was approved by the hospital institutional review board for Malaysian HIV-infected patients (MEC 975.6). |

Note that full information on the approval of the study protocol must also be provided in the manuscript.

## Flow Cytometry

### Plots

Confirm that:

- [x] The axis labels state the marker and fluorochrome used (e.g. CD4-FITC).
- [x] The axis scales are clearly visible. Include numbers along axes only for bottom left plot of group (a 'group' is an analysis of identical markers).
- [x] All plots are contour plots with outliers or pseudocolor plots.
- [x] A numerical value for number of cells or percentage (with statistics) is provided.

### Methodology

| | |
|---|---|
| Sample preparation | Informations are provided in the methods section |
| Instrument | FACS: BD Symphony, BD Fortessa, BD LSR II |
| Software | DIVA, FlowJo, R studio, Cytofkit software. |
| Cell population abundance | The purity of the post-fraction was systematically measured by flow cytometry on BD LSR II. The purity of the sorted population was above 95 %. |
| Gating strategy | Information available in Fig S1 |

- [x] Tick this box to confirm that a figure exemplifying the gating strategy is provided in the Supplementary Information.
