# Supplementary material for: Immunological history governs human stem cell memory CD4 heterogeneity via the Wnt signaling pathway
Source: Nat Commun. 2020 Feb 10;11:821. doi: 10.1038/s41467-020-14442-6 (PMC7010798; doi:10.1038/s41467-020-14442-6)
Supplement: Supplementary file 4 — Description of Additional Supplementary Files [file 41467_2020_14442_MOESM4_ESM.pdf]

Description of Additional Supplementary Files

Supplementary Data 1

Pathway analysis of CD4 T_{SCM} clusters during aging
